# Simulation Curriculum Improves Emergency Medicine Resident Preparedness for the New American Board of Emergency Medicine Certifying Exam

**DOI:** 10.5811/westjem.48651

**Published:** 2026-01-03

**Authors:** Ian Batson, Chinezimuzo Ihenatu, Frances Shofer, Matthew Magda, Michael E. Abboud, Lauren Conlon, Suzana Tsao, Mira Mamtani

**Affiliations:** * University of Pennsylvania, Department of Emergency Medicine, Philadelphia, Pennsylvania; † University of Pennsylvania, Department of Epidemiology and Biostatistics, Philadelphia, Pennsylvania

## Abstract

**Introduction:**

In 2024, the American Board of Emergency Medicine (ABEM) announced the launch of a new certifying exam that emergency medicine (EM) residency graduates must pass to achieve specialty certification. To date, there are no comprehensive curricula published in the available literature to aid residents in exam preparation.

**Methods:**

In this pre-post pilot study, 44% (24/55) of postgraduate year 1 (PGY-1) through PGY-4 EM residents at a single site participated in a four-hour simulated certifying exam curriculum. Learners were asked to complete a four-point Likert scale survey rating self-reported preparedness (very unlikely – very likely) to take the ABEM Certifying Exam, as well as comfort with the ABEM tested competencies, preceding and following the simulation session.

**Results:**

Survey respondents (n = 21; 87.5%) reported an improvement in overall preparedness to take the ABEM Certifying Exam, yielding a pre-post mean difference score of +1.2 (1.9 [unlikely] pre to 3.1 [likely] post, P < .001). Additionally, there was an improvement in all ABEM-tested competencies; pre-post mean difference score ranged from +0.5 (3.0 pre to 3.5 post) for patient-centered communication to +1.1 (2.2 pre to 3.3 post) for clinical decision-making (P < .001 for all competencies).

**Conclusion:**

Given the critical need, and self-reported improvement in preparedness, EM training programs nationwide could consider incorporating a similar simulation curriculum into their didactic experience to help better prepare their learners for the new ABEM Certifying Exam.

## INTRODUCTION

Every year, over 2,500 physicians achieve American Board of Emergency Medicine (ABEM) certification, indicating that they have demonstrated sufficient knowledge and clinical skills to provide competent patient care.^1^ Obtaining ABEM certification entails the completion of a two-step process comprised of a written (qualifying) exam followed by a practical exam. This practical exam, known as the “oral exam,” was instituted over 45 years ago and consisted of seven scenario-based interviews meant to measure skills otherwise challenging to assess in a multiple-choice format. In February 2024, ABEM announced the release of a new practical exam, the ABEM Certifying Exam, which will replace the oral exam in 2026. The new certifying exam focuses on the following competencies: difficult conversations; managing conflict; patient- centered communication; reassessment/troubleshooting; procedures; ultrasound; clinical decision-making; and prioritization.^2^

Pre-existing published resources to guide exam preparation are limited. Therefore, there is a critical need to create curricula that prepare emergency medicine (EM) residents for the new ABEM Certifying Exam. One tool that has been used successfully in other domains is medical simulation. A systematic review of simulation-based medical education found that simulation, when paired with traditional didactic education, has a superior impact on confidence, knowledge retention, and clinical performance compared to didactic education alone.^3^ Furthermore, prior studies revealed that medical simulation leads to improved Objective Structured Clinical Examination (OSCE) performance among medical students and residents.^4^–^6^ Medical simulation has also been found to have a favorable effect on assessment of interpersonal communication competence.^6^ A four-day immersive simulation curriculum yielded comparable OSCE performance to a six-week clinical rotation in cardiology and respiratory medicine.^7^ Simulation has also had a favorable impact on skill acquisition, such as ultrasound and medical procedures.^4^,^8^ Lastly, medical simulation resulted in improved confidence and decreased anxiety preceding summative exams.^9^

While prior studies suggest that simulation may be helpful in preparing residents for future examinations, there is currently no published data evaluating the impact of simulation curricula on preparing EM residents for the new ABEM Certifying Exam. In this pilot study, we assessed the effect of a novel simulation curriculum on resident self-reported preparedness for the ABEM Certifying Exam. The included cases can be easily replicated by other EM residency programs to aid their residents in exam preparation.

## METHODS

In this pre-post pilot study, we invited all EM residents at a single, urban EM training program to participate in a simulated ABEM Certifying Exam curriculum. Our primary outcome was change in resident self-reported preparedness for the exam before and after the simulated curriculum, as measured by surveys distributed to learners preceding and following the four-hour curriculum.

### Survey Development

We developed pre- and post-simulation surveys using an iterative process with the intention to create easy-to-understand questions stems, omitting double-barreled questions, implicit negatives, and leading questions. Survey questions were meant to assess level 1 (reaction) and level 2 (learning) of the Kirkpatrick model.^[10]^ We conducted pilot testing on attending physicians not included within the studied population. The pre-simulation survey included nine questions, which were intended to evaluate trainees’ overall perceived level of preparedness to take the exam, as well as their comfort with individual competencies: difficult conversations; managing conflict; patient-centered communication; reassessment/troubleshooting; procedures; ultrasound; clinical decision-making; and prioritization (Appendix 1). Each question was graded on a four-point Likert scale (1=very unlikely, 2=unlikely, 3=likely, 4=very likely) on the trainees’ confidence in passing the ABEM Certifying exam right now. We chose a four-point Likert scale to force a non-neutral response. A similar survey was delivered at the close of the simulation session (Appendix 1), which was identical to the pre-simulation survey aside from the inclusion of two additional questions evaluating the effectiveness of the curriculum and overall suggestions for improvement.

### Curriculum Description

This curriculum was designed using Kern’s six-step model for curriculum development.^11^ There is a general need among EM training programs to help prepare learners for the new ABEM Certifying Exam, but there are limited resources available for preparation. Within our program, we identified a targeted need among our senior residents who will be among the initial cohort across the country taking this exam. The overarching goal in developing this curriculum was to prepare our residents for the overall format and the individual competencies tested for the ABEM Certifying Exam.

Educational strategies used simulation to replicate the structure of the Certifying Exam. Several learning theories support the use of simulation as an appropriate modality to help prepare learners for this new ABEM requirement, including behaviorally oriented experiential learning, which posits that knowledge gained from confronting a problem in a simulated environment can be applied to real-world situations, and reflection on action, which suggests feedback obtained during the simulation debriefing will trigger self-reflection on performance and guide further efforts for exam preparation.^12^ Implementation required obtaining buy-in from program leadership, faculty, and trainees. Finally, program evaluation is ongoing and includes a post-simulation survey of participants.

In total, this curriculum took place over the course of four hours. Residents first attended a lecture including a description of, and grading criteria for, the four case types assessing interpersonal communication. Participants then rotated through a series of four stations where one resident acted as the examinee for each while the others observed. They then received immediate feedback on their performance from the facilitator and their colleagues. Participants then received a second lecture on technical skills and clinical reasoning case types, followed by practice sessions with each case type in a similar format to the interpersonal communication stations. The session concluded with a post-simulation survey.

Simulation cases were written to mimic those on the ABEM Certifying Exam based on publicly available resources on the ABEM website. These included four cases assessing interpersonal communication (difficult conversations, managing conflict, patient-centered communication, and reassessment/troubleshooting), two cases evaluating technical skills (ultrasound and procedures), and two cases assessing clinical reasoning (clinical decision-making and prioritization). Interpersonal communication and ultrasound cases were led by two faculty members, one playing the role of a mock patient and the other acting as a facilitator. The remaining cases required only one faculty member to act as a facilitator. Case materials varied by case type and are included as supplementary material.

### Data Collection

We collected survey data anonymously, although each participant created a unique, non-identifiable code to pair the pre- and post-simulation results. There were no surveys with incomplete or missing data, although pre-simulation surveys without corresponding post-simulation data were excluded from analysis. To determine differences in the nine survey questions pre/post-simulation, we performed paired *t*-tests. To adjust for multiple comparisons, *P*-values < .01 were considered statistically significant. Differences pre/post are reported as means with 95% confidence intervals. All analyses were performed using SAS statistical software v9.4 (SAS Institute, Inc, Cary, NC). Figures were created using GraphPad Prism v10.4.1 (GraphPad Software, San Diego, CA). The University of Pennsylvania Institutional Review Board reviewed our study and deemed it exempt.

## RESULTS

Of the 55 PGY-1 to PGY-4 EM residents invited to attend, 24 (43.6%) elected to participate. This included four PGY-1, eight PGY-2, four PGY-3, and eight PGY-4 residents. Of the 24 in attendance, 21 (87.5%) completed both the pre- and post-surveys. All group comparisons reached statistical significance. Overall confidence pre-simulation was low, with five (24%) residents reporting that they were likely to very likely to pass the ABEM Certifying Exam (Table 1). Post simulation, 17 (81%) respondents reported that they were likely to very likely to pass, yielding a 57% improvement (Table 1). Overall confidence to pass the ABEM Certifying Exam increased by 1.2 points from an average of 1.9 to 3.1 (unlikely to likely) on a four-point Likert scale (95% CI, 0.92–1.46, *P* < .001, Figure).

Similarly, across all competencies, we observed increases in self-reported learner preparedness. Mean improvements in pre/post scores ranged from +0.47 (patient-centered communication) to +1.1 (clinical decision-making, Figure) and percentage increases in confidence ranging from 9.5–47% (Table). Individual group comparisons are as follows:

Clinical reasoning stations: clinical decision-making scores increased by 1.1 points from an average of 2.2 to 3.3. Prioritization scores increased by 0.7 points from an average of 2.4 to 3.1

### Technical skills stations

Ultrasound scores increased by 0.8 points from an average of 2.6 to 3.4. Procedures scores increased by 0.5 points from an average of 2.8 to 3.3.

### Interpersonal communication stations

Difficult conversations scores increased by 0.7 points from an average of 2.8 to 3.5. Managing conflict scores increased by 0.8 points from an average of 2.6 to 3.4. Patient-centered communication scores increased by 0.5 points from an average of 3.0 to 3.5. Lastly, reassessment and troubleshooting scores increased by 0.7 points from an average of 2.6 to 3.3.

Overall satisfaction with this curriculum was favorable with 95% of respondents (20/21) agreeing (n=6) or strongly agreeing (n=14) that they felt more prepared to take the ABEM Certifying Exam following this curriculum. The one participant who strongly disagreed with this statement commented, “super-helpful, thank you for setting this up,” raising the possibility of an erroneous entry. Respondents requested that a list of possible procedures and ultrasound exams that could be tested in the ABEM Certifying Exam be highlighted in the introductory presentation.

## DISCUSSION

To our knowledge, this is the first study to report on a simulation curriculum that prepares residents to take the 2026 ABEM Certifying Examination and helps fill a critical gap in exam-preparatory resources. We observed a positive correlation between completion of a simulated exam and self-rated preparedness to take the certifying exam both overall and within each of the eight tested competencies. Furthermore, most residents rated that they felt more prepared to take the certifying exam as a direct result of this curriculum.

The practice of EM has changed since the inception of the oral board exam in 1980. As a paternalistic approach to medicine has fallen out of favor, the importance of interpersonal communication has grown. In the age of shared decision-making and patient advocacy, patient-centered communication, managing conflict, and the companionate delivery of sensitive information have become increasingly important skills. Likewise, given increasing emergency department boarding times, effective reassessment and troubleshooting have become critical facets of daily practice. Lastly, given the widespread uptake of point-of-care ultrasound among emergency clinicians, demonstrating proficiency with this modality is essential. Therefore, the replacement of the traditional oral board exam with the new certifying exam is reflective of the contemporary practice of emergency medicine.

In addition, with the rapid expansion and use of artificial intelligence (AI) in medical education, learners have access to medical knowledge with far more ease than in prior decades. This raises the question of what competencies might be integral to the effective practice of EM in the future. One could argue that the competencies chosen by ABEM, such as procedures and difficult conversations, should be taught and assessed in a more comprehensive manner in EM training. For example, in the clinical learning environment, one could imagine that when a learner is questioned about a specific disease process to assess knowledge, they could use AI to rapidly provide an answer. Or while a resident is providing bedside care, AI could help generate differential diagnoses and management plans. Teaching and assessing these other skills highlighted by the ABEM Certifying Exam, such as managing interdisciplinary conflict and patient-centered communication, could help residents expand their skills to those that would be complementary to AI and critical to the effective practice of EM in the future. Our study identifies an increase in confidence in these types of competencies among participants. Further efforts could be directed into expansion of this curriculum to meet these anticipated future needs.

While the content of the exam mirrors contemporary residency training and may be informative to the future practice of EM, there is a lack of resources to aid in preparation. The existing oral board exam has a 96% pass rate, which is largely attributable to an abundance of existing courses, residency didactic curricula, and resources for independent practice.^1^ Given that no such resources are available for the new certifying exam, there is reasonable concern among EM residents over the possibility of declining board pass rates. While it remains to be seen whether an effect on pass rates will come to fruition, simulation curricula intended to mimic summative exams increase confidence and decrease test-taking apprehension, which in turn has shown a positive impact on resident wellbeing.^9^ Our study highlights the overall improved confidence learners have in passing the ABEM Certifying Exam following this curriculum. Our study team will follow this cohort to obtain longer term data, including preparedness at the time of graduation. We also plan to compare ABEM Certifying Exam pass rate among those who participated compared to those who did not participate in this simulated curriculum.

## LIMITATIONS

While learners noted that they felt more prepared to take the ABEM Certifying Exam, longer term studies are needed to measure board pass rate following the implementation of a certifying-exam simulation curriculum. This is especially critical as our current cohort of residents has no prior experience with the new exam and, therefore, perceived preparedness may not translate into improved board pass rates. Additionally, given that participation in this curriculum was voluntary, with a subset of residents choosing to participate, there is the potential for selection bias. This also led to a modest sample size, which increases the risk of sampling bias and thus reduces power. Future iterations of this curriculum will include additional members of our residency program.

Moreover, the single-site design of this pilot study may limit generalizability; however, the provided curriculum could be easily adopted or adapted at other training programs interested in preparing their learners for this new exam. Additionally, we were unable to analyze the effect by PGY level. Future studies could explore whether a curriculum like this would be better suited for a more junior vs senior learner. Finally, while we used parametric statistics to analyze ordinal data, results were similar using non-parametric statistics, and prior studies support the statistical equivalence of using parametric and non-parametric testing when analyzing Likert scales.^13^

## CONCLUSION

Our simulated certifying exam curriculum, the first of its kind to be described in the literature, can help meet a critical need for exam preparation resources among EM residents. With the cases created for this study, other training programs can design a simulated ABEM Certifying Exam experience for their trainees with minimal resources to better prepare their residents for this requirement. Future studies should explore alternative tools and curricula to better teach and assess EM residents for the new competencies critical for the ABEM Certifying Exam, as well as the practice of emergency medicine now and in the future.

## Supplementary Information



## Figures and Tables

**Figure:**
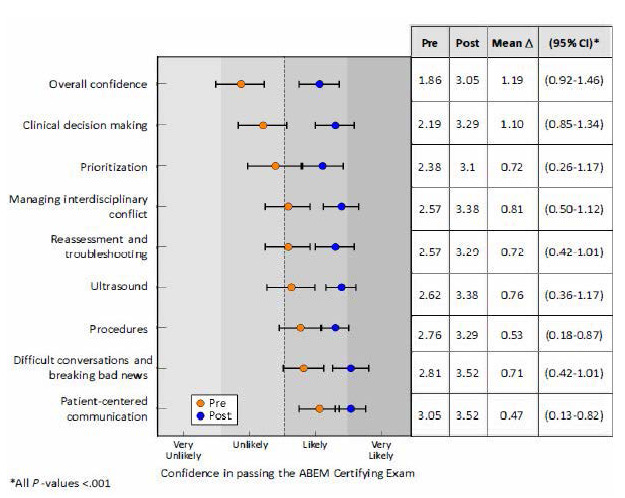
In a study measuring perceived exam preparedness among emergency medicine residents following a simulated ABEM Certifying Exam, comparison of mean differences in self-reported learner preparedness scores on pre- vs post-simulation surveys. *ABEM*, American Board of Emergency Medicine.

**Table:** In a study measuring perceived exam preparedness among emergency medicine residents following a simulated ABEM Certifying Exam, comparison of the percentage of participants responding “likely” or “very likely” to pass on pre- vs post-simulation surveys.

	Absolute % likely or very likely		Difference: Post- pre

	Pre	Post	
Overall confidence	23.8%	81.0%	57.2%
Clinical decision-making	42.9%	90.5%	47.6%
Managing conflict	61.9%	95.2%	33.3%
Ultrasound	52.4%	100.0%	47.6%
Prioritization	47.6%	81.0%	33.4%
Reassessment and troubleshooting	61.9%	90.5%	28.6%
Difficult conversation	76.2%	95.2%	19.0%
Procedures	71.4%	100.0%	28.6%
Patient-centered communication	90.5%	100.0%	9.5%

*ABEM*, American Board of Emergency Medicine.
