# Supplementary material for: Simulation Curriculum Improves Emergency Medicine Resident Preparedness for the New American Board of Emergency Medicine Certifying Exam
Source: West J Emerg Med. 2026 Jan 3;27(1):39–43. doi: 10.5811/westjem.48651 (PMC12815499; doi:10.5811/westjem.48651)
Supplement: Supplementary file 1 [file wjem-27-39-s001.docx]

**Appendix 1**

**Pre and Post Questions:**

If you were to take the ABEM qualifying exam (oral board exam) right now, how confident do you feel that you would pass? (4 point Likert scale)

| Very Unlikely | Unlikely | Likely | Very Likely |
| --- | --- | --- | --- |
| ⭘ | ⭘ | ⭘ | ⭘ |

If you were to take the ABEM qualifying exam (new oral board exam) right now, how confident do you feel that you would pass the following case types individually?

|  | Very Unlikely | Unlikely | Likely | Very Likely |
| --- | --- | --- | --- | --- |
| Clinical decision making | ⭘ | ⭘ | ⭘ | ⭘ |
| Prioritization | ⭘ | ⭘ | ⭘ | ⭘ |
| Ultrasound | ⭘ | ⭘ | ⭘ | ⭘ |
| Procedures | ⭘ | ⭘ | ⭘ | ⭘ |
| Difficult conversations | ⭘ | ⭘ | ⭘ | ⭘ |
| Managing conflict | ⭘ | ⭘ | ⭘ | ⭘ |
| Patient-centered communication | ⭘ | ⭘ | ⭘ | ⭘ |
| Reassessment and troubleshooting | ⭘ | ⭘ | ⭘ | ⭘ |

**Post Only**

Do you feel more prepared to take the ABEM certifying exam as a result of today's session? (4-point Likert Scale) strongly disagree – strongly agree

| Strongly Disagree | Disagree | Agree | Strongly Agree |
| --- | --- | --- | --- |
| ⭘ | ⭘ | ⭘ | ⭘ |

Pre

<https://upenn.co1.qualtrics.com/survey-builder/SV_9SmEbFjG8qZejRQ/edit>

Post

<https://upenn.co1.qualtrics.com/survey-builder/SV_4N34tZEWiTyoZam/edit>
